# Association Between Asthma and the Risk of Type 2 Diabetes Mellitus: Results From NHANES 2009–2016 Data

**DOI:** 10.1155/ije/4046954

**Published:** 2026-01-31

**Authors:** Jinyue Meng, Decheng Lu, Jianli Huang, Li Liu, Cen Huang, Jinqun Ye, Xuemei Huang

**Affiliations:** ^1^ Department of Endocrinology, The First People’s Hospital of Nanning, Nanning, Guangxi, 530022, China; ^2^ Department of Endocrinology, The Fifth Affiliated Hospital of Guangxi Medical University, Nanning, Guangxi, 530022, China, gxmu.edu.cn; ^3^ Department of Endocrinology, The First Affiliated Hospital of Guangxi Medical University, Nanning, Guangxi, 530021, China, gxmu.edu.cn

**Keywords:** asthma, NHANES, risk factor, Type 2 diabetes mellitus

## Abstract

**Objective:**

This study aimed to investigate the relationship between asthma and the risk of Type 2 diabetes mellitus (T2DM) through a cross‐sectional analysis of the National Health and Nutrition Examination Survey (NHANES) data from 2009 to 2016.

**Methods:**

Weighted *t*‐tests and weighted chi‐square tests were used to compare the baseline characteristics between patients with T2DM and individuals without T2DM. Weighted multivariate logistic regression models were used to determine the association between asthma and the risk of T2DM. Two‐sample univariate Mendelian randomization (MR) was performed to analyze asthma and the risk of T2DM.

**Results:**

Among the 2348 participants included, the prevalence of asthma was 70.9% in T2DM patients. The results of the weighted multivariate logistic regression models revealed that asthma was significantly positively linked to T2DM risk, with odds ratios of 2.24, 2.26, and 1.92 in Models 1, 2, and 3, respectively. The fitting curve analysis demonstrated that asthma was positively correlated with the risk of T2DM. The MR results revealed a marked causal effect of asthma on T2DM, identifying asthma as a risk factor for T2DM. Sensitivity analysis confirmed the robustness of MR findings.

**Conclusion:**

Asthma was significantly and positively associated with T2DM risk, indicating that it serves as a risk factor for the onset of this condition.

## 1. Introduction

Diabetes mellitus (DM) is one of the most prevalent chronic diseases worldwide and is characterized by a clinical syndrome of hyperglycemia. The prevalence of DM has surged dramatically over the past few decades, with an increasing incidence of Type 2 DM (T2DM), making it one of the most pressing public health issues. The number of individuals diagnosed with DM is projected to reach 350 million by 2030 [1, 2]. Individuals with DM have an elevated risk of DM‐related complications, including cardiovascular diseases, retinopathy, nephropathy, and neuropathy. These conditions significantly impair health quality and overall life satisfaction. DM is currently the seventh leading cause of mortality worldwide [3–5]. Existing therapeutic options, such as insulin injections and oral hypoglycemic agents, control blood glucose levels but do not prevent the development of DM [6, 7]. Consequently, addressing the increasing prevalence of DM requires targeted screening to identify those who are at risk.

Asthma arises from a complex interplay between environmental and genetic factors and is characterized by chronic inflammation and airway hyperresponsiveness. A systematic review has suggested a potential bidirectional relationship between asthma and T2DM [8]. Thomsen et al. [9] found that patients with T2DM have an increased risk of developing asthma compared to individuals without T2DM. In addition, a survey indicated that adolescents with active asthma were at a heightened risk of T2DM [10]. Another study indicated that individuals with T2DM were less likely to develop asthma, suggesting a potential protective effect of T2DM against the onset of asthma [11]. Baek et al.’s nationwide study in Korea revealed that T2DM without retinopathy was not associated with an increased risk of asthma [12]. Currently, methodological heterogeneity or small sample sizes hinder definitive conclusions regarding publication bias. Therefore, the association between asthma and T2DM remains ambiguous.

The National Health and Nutrition Examination Survey (NHANES) is a comprehensive national study conducted by the U.S. Centers for Disease Control and Prevention. It employs structured interviews, health screenings, and laboratory sample analyses to gather data from participants [13, 14]. To elucidate the association between asthma and T2DM, we performed a retrospective analysis using data from the NHANES conducted between 2009 and 2016 among adults and further assessed the causality between asthma and the risk of T2DM using Mendelian randomization (MR).

## 2. Materials and Methods

### 2.1. Study Design and Participants

**FIGURE 1:**
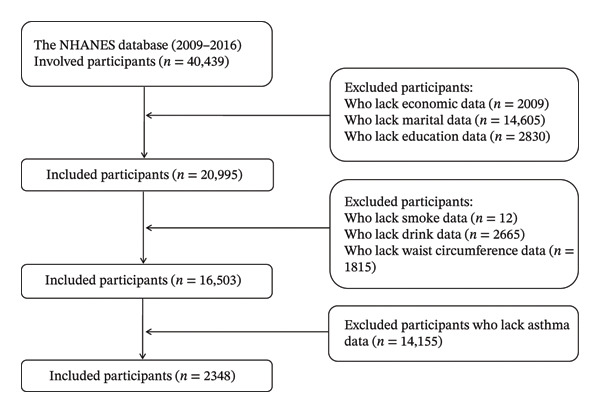
Exclusion criteria for subjects participating in the NHANES survey.

This study used cross‐sectional data collected from the NHANES database between 2009 and 2016. All study participants provided written informed consent, and the study protocol was approved by the National Center for Health Statistics Research Ethics Review Committee (Approval number: #2005‐06, #2011‐17). The initial cohort comprised 40,439 adults; however, individuals lacking economic data (*n* = 2009), marital status information (*n* = 14,605), educational background details (*n* = 2830), smoking history (*n* = 12), alcohol consumption records (*n* = 2665), waist circumference measurements (*n* = 1815), and asthma‐related data (*n* = 14,155) were excluded. Ultimately, this analysis included 2348 subjects, as depicted in Figure 1.

### 2.2. Assessment of Variables

Participants who met one of the following criteria were defined as T2DM: (1) self‐reported diagnosis by a physician, (2) fasting plasma glucose levels ≥ 7.0 mmol/L, (3) 2‐h plasma glucose levels during an oral glucose tolerance test ≥ 11.1 mmol/L, (4) hemoglobin A1c levels ≥ 6.5%, or (5) use of oral hypoglycemic agents or insulin therapy. Asthma status was assessed using the following MCQ 035 item: “Do you still have asthma?” Individuals who responded with “yes” were categorized as the asthma group, while those who answered “no” were classified as the nonasthmatic group. Confounding factors included age, sex, body mass index (BMI), waist circumference, race, marital status, household income, smoking status, physical activity level, alcohol consumption, blood pressure, hemoglobin concentration, and alanine transaminase, glutamate transpeptidase, serum creatinine, and serum uric acid levels. Smoking status was classified as follows: individuals who smoked no more than 100 cigarettes in their lifetime were considered nonsmokers, while those who smoked more than 100 cigarettes in their lifetime were classified as smokers. Drinking status was defined as follows: individuals who drank fewer than 12 times a year were considered nondrinkers, whereas those who drank 12 or more times a year were classified as drinkers.

### 2.3. Association Analysis of Asthma With T2DM Risk

Multivariate logistic regression models were used to elucidate the association between asthma and T2DM by calculating odds ratios (ORs) and 95% confidence intervals (CIs). Three models were constructed: Model 1 was not adjusted for covariates; Model 2 was adjusted for age, race, and sex only; and Model 3 was adjusted for all identified covariates. The relationship between asthma and T2DM was further evaluated using fitting curves.

### 2.4. MR Analysis

We conducted two‐sample MR analyses using data from previous genomewide association studies (GWAS). Genetic variant data for asthma and T2DM were obtained from a GWAS analysis performed by the Integrated Epidemiology Unit (IEU) Open‐GWAS (https://gwas.mrcieu.ac.uk/). The GWAS ID ukb‐b‐11411 for asthma contained 9,851,867 single‐nucleotide polymorphisms (SNPs) from 462,013 European populations (9440 asthma cases and 452,573 control samples), and the GWAS ID ebi‐a‐GCST006867 for T2DM contained 5,030,727 SNPs from 655,666 European populations (61,714 T2DM cases and 1178 control samples). SNPs associated with asthma were selected as exposure data by applying a significance threshold of *p* < 5 × 10^−8^. Using the European 1000 Genomes dataset to assess linkage disequilibrium (LD), we further screened for SNPs that were not in LD; the cutoff for LD was set at *r* ^2^ < 0.001. F‐statistics were calculated to evaluate the strength of the SNPs, with an *F*‐value greater than 10 indicating a strong instrument. Five algorithms were employed for MR analysis concerning the impact of asthma on T2DM risk: MR‐Egger regression, weighted median estimation, inverse variance weighting (IVW), simple mode estimation, and weighted mode estimation. The results primarily referenced the IVW estimates. Sensitivity analyses were conducted to ensure robustness of the MR estimates.

### 2.5. Statistical Analysis

Data analysis was conducted using the survey package in the R statistical software (Version R 4.2.1, macOS). Continuous variables are presented as mean ± standard deviation, while categorical variables are expressed as weighted percentages (%). Weighted *t*‐tests (continuous variables) or weighted chi‐square tests (categorical variables) were used to compare the differences in baseline characteristics between participants with and without T2DM, as well as the differences in baseline characteristics among the quartile groups of exposure factors. To further study the influence of covariates on this association, a weighted multivariate logistic regression model was used to explore the relationship between asthma and T2DM, and the adjusted ORs and 95% CIs were calculated. This model is divided into Model 1 (unadjusted), Model 2 (incompletely adjusted), and Model 3 (fully adjusted). Furthermore, to explore whether the conclusions of different populations were consistent, subgroup analyses were conducted to categorize variables such as age, sex, race, education level, smoking status, drinking status, hypertension, BMI, physical activity, and waist circumference to evaluate the adjustment effect. A two‐sample MR analysis was performed with T2DM as the outcome variable and asthma as the exposure variable. Five methods were used to assess the causal relationship between asthma and T2DM: MR‐Egger, weighted median, IVW, simple mode, and weighted mode. Heterogeneity and directional pleiotropy were evaluated using the Cochrane *Q* test and Egger’s intercept test, respectively. Sensitivity analysis was performed using the leave‐one‐out approach. A two‐sided *p* value of < 0.05 was deemed statistically significant.

## 3. Results

### 3.1. Participant Characteristics

This study included 2348 participants, comprising 395 individuals with T2DM and 1953 individuals without T2DM. Stratified by asthma status, the cohort consisted of 280 individuals with T2DM and asthma, 115 with T2DM but without asthma, 1069 individuals with asthma but without T2DM, and 884 without either condition. The prevalence of asthma was 70.9% among patients with T2DM and 54.7% among participants with T2DM. Among those diagnosed with T2DM, it was observed that 69.6% had a BMI ≥ 30; in addition, 86.3% were classified as abdominally obese, while smoking prevalence stood at 57.5%. Furthermore, only a minority (32.9%) engaged in sufficient physical activity, 65.6% were drinkers, and a significant proportion (74.2%) had hypertension. There were significant differences in BMI, waist circumference, smoking habits, physical activity levels, and blood pressure between patients with T2DM and individuals without T2DM (*p* < 0.05). Moreover, compared to participants without T2DM, those with T2DM exhibited lower hemoglobin levels, elevated alanine transaminase levels, and increased glutamate transpeptidase levels; serum creatinine and uric acid concentrations were also higher (*p* < 0.05) (Table 1).

**TABLE 1:** Clinical and biochemical characteristics of patients with T2DM and individuals without T2DM.

Characteristics	T2DM (*n* = 395)	Non‐T2DM (*n* = 1953)	*p* value
Age, years (%)	51.02 (7.90)	50.84 (8.19)	0.911
Gender (%)			
Male	157 (39.7)	833 (42.7)	0.312
Female	238 (60.3)	1120 (57.3)	
BMI, kg/m^2^ (%)			
< 25	33 (8.4)	570 (29.2)	< 0.001
25–30	87 (22.0)	592 (30.3)	
≥ 30	275 (69.6)	791 (40.5)	
Waist circumference (%)			
Normal	54 (13.7)	833 (42.7)	< 0.001
Obesity	341 (86.3)	1120 (57.3)	
Race (%)			
Mexican American	49 (12.4)	176 (9.0)	< 0.001
Non‐Hispanic Black	107 (27.1)	433 (22.2)	
Non‐Hispanic White	146 (37.0)	969 (49.6)	
Other race	93 (23.5)	375 (19.2)	
Marital status (%)			
Cohabitated	258 (65.3)	1147 (58.7)	< 0.001
Solitary	137 (34.7)	806 (41.3)	
Economic (%)			
Low income	173 (43.8)	712 (36.5)	< 0.001
Medium income	157 (39.7)	654 (33.5)	
High income	65 (16.5)	587 (30.1)	
Smoking status (%)			
Smoker	227 (57.5)	922 (47.2)	< 0.001
Nonsmoker	168 (42.5)	1031 (52.8)	
Physical activity (%)			
Insufficient exercise	265 (67.1)	912 (46.7)	< 0.001
Moderate exercise	101 (25.6)	505 (25.9)	
Intensive exercise	29 (7.3)	536 (27.4)	
Drinking status (%)			
Drinker	259 (65.6)	1509 (77.3)	< 0.001
Nondrinker	136 (34.4)	444 (22.7)	
Hemoglobin (g/L)	69.21 (16.58)	73.85 (14.83)	< 0.001
Hypertension (%)	293 (74.2)	648 (33.2)	< 0.001
Alanine transaminase (U/L)	23.52 (19.28)	20.88 (16.57)	0.005
Glutamate transpeptidase (U/L)	33.37 (34.78)	23.81 (28.56)	< 0.001
Serum creatinine (umol/L)	63.51 (35.59)	55.83 (23.51)	< 0.001
Serum uric acid (umol/L)	44.31 (16.09)	40.75 (13.77)	< 0.001
MCQ 035 (%)			
Asthma	280 (70.9)	1069 (54.7)	< 0.001
Nonasthma	115 (29.1)	884 (45.3)	

Abbreviations: BMI, body mass index; SD, standard deviation; T2DM, Type 2 diabetes mellitus.

### 3.2. Association Between Asthma and T2DM

**FIGURE 2:**
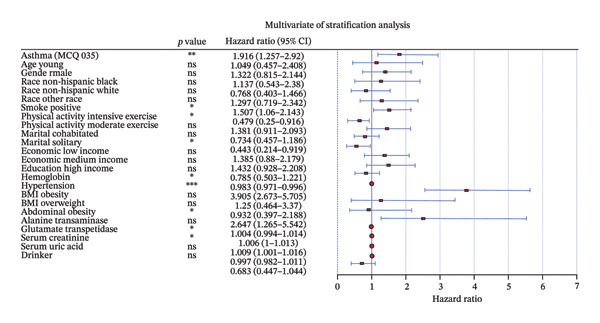
Weighted logistic regression analysis of asthma and the risk of T2DM.

To analyze the association between asthma and the risk of developing T2DM, we constructed multivariate logistic regression models. The ORs were 2.24 (95% CI = 1.55–3.24, *p* < 0.05), 2.26 (95% CI = 1.55–3.31, *p* < 0.05), and 1.92 (95% CI = 1.26–2.92, *p* < 0.05) in Models 1, 2, and 3, respectively (Table S1). In addition, we performed risk stratification analysis which revealed a significant association between asthma and T2DM within specific subgroups including smoking group (OR = 1.507, 95% CI = 1.060–2.143, *p* < 0.05), physical activity intensive exercise group (OR = 0.479, 95% CI = 0.250–0.916, *p* < 0.05), hypertension group (OR = 3.905, 95% CI = 2.673–5.705, *p* < 0.05), and waist circumference obesity group (OR = 2.647, 95% CI = 1.265–5.542, *p* < 0.05) in Model 3 (Figure 2). The fitting curve showed a positive correlation indicating that asthma was positively associated with an increased risk of T2DM (Figure 3).

FIGURE 3Analysis of the relationship between asthma and T2DM. (a) Fitting curve analysis of asthma and T2DM. (b) Forest plot of the causal relationship between asthma and T2DM. (c) Funnel plot of the causal relationship between asthma and T2DM.

**Figure:**
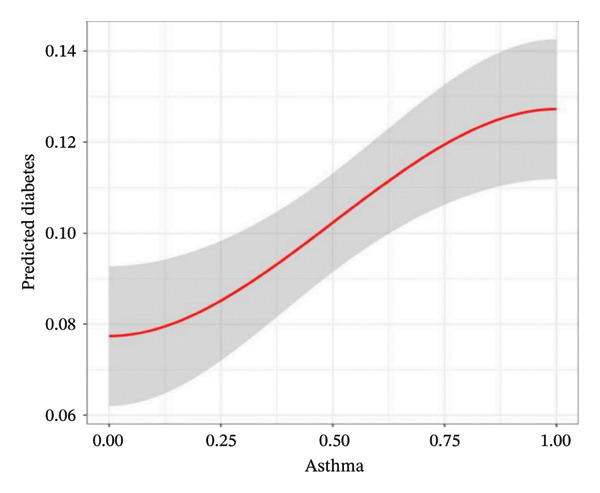
(a)

**Figure:**
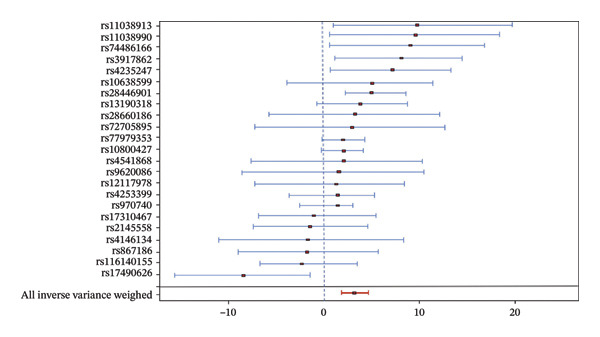
(b)

**Figure:**
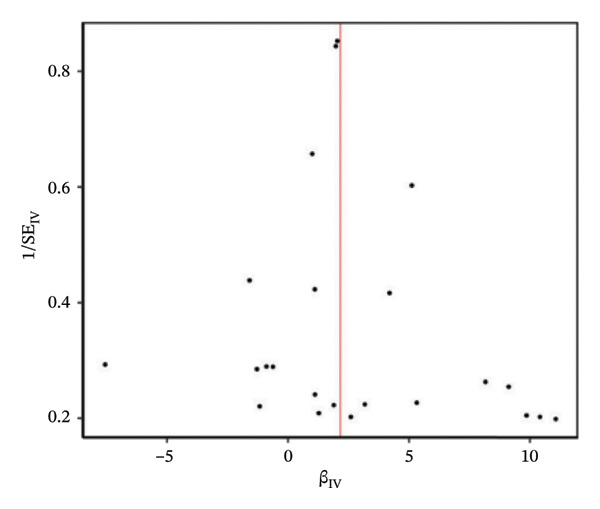
(c)

### 3.3. Causal Relationship Between Asthma and the Risk of T2DM

An MR study was conducted to investigate the causal relationship between asthma and the risk of T2DM. After rigorous screening, 23 single SNPs were identified. The MR results revealed that asthma had a significant causal effect on the risk of T2DM, as determined using the IVW method (Table 2). The MR effect value depicted in the forest plot was > 0, indicating that asthma has a positive effect on T2DM. According to the funnel plot, the data distribution is uniform and relatively symmetrical on the left and right sides, which conforms to Mendel’s second law of independent assortment (Figure 3). Cochran’s *Q* test indicated no substantial heterogeneity in the effect of asthma on T2DM (Table 3). Furthermore, the pleiotropy test demonstrated the absence of horizontal pleiotropy in the MR estimates (*p* = 0.064). The results of the leave‐one‐out sensitivity analysis showed that no individual SNP produced a sensitive outcome, confirming that the causal relationship between asthma and T2DM risk remained unaffected by any single SNP.

**TABLE 2:** MR analysis results between asthma and T2DM risk.

Outcome	Exposure	Method	SNP (n)	OR	*p* value
T2DM	Asthma	MR‐Egger	23	4.976	0.1514
T2DM	Asthma	Weighted median	23	7.315	0.0075
T2DM	Asthma	Inverse variance weighted	23	8.754	0.0007
T2DM	Asthma	Simple mode	23	3.666	0.3263
T2DM	Asthma	Weighted mode	23	5.948	0.0235

Abbreviations: MR, Mendelian randomization; OR, odds ratio; SNP, single‐nucleotide polymorphism; T2DM, Type 2 diabetes mellitus.

**TABLE 3:** Heterogeneity test results.

Outcome	Exposure	Method	Q *p* value
T2DM	Asthma	MR‐Egger	0.0518
T2DM	Asthma	Inverse variance weighted	0.0593

Abbreviation: MR, Mendelian randomization; T2DM, Type 2 diabetes mellitus.

## 4. Discussion

This inconsistency of previous research results prompted us to analyze the relationship between asthma and T2DM. We analyzed the causal relationship between asthma and the risk of T2DM through NHANES data and MR analysis. The results of this study indicate a significant positive association between asthma and T2DM risk and establish asthma as a risk factor for T2DM. A survey conducted as part of the Singapore Chinese Health Study revealed that adults with asthma were at a higher risk of developing DM than children with asthma [15]. Chronic systemic inflammatory responses have been implicated in the progression of asthma and T2DM [16, 17]. A prospective cohort study demonstrated that asthma was independently associated with an increased risk of T2DM, suggesting that chronic airway inflammation may play a role in the pathogenesis of T2DM [18]. Gulcan et al. [19] indicated that disturbances in glucose metabolism due to inflammation‐induced insulin resistance may occur in patients with asthma, thereby elevating the risk of T2DM. Yun et al. [20] found that asthma may increase the proinflammatory polarization of helper T cells in individuals with T2DM. Zhan et al. [21] MR analyses, employing the IVW‐RE/IVW method, revealed a statistically significant association between asthma and T2DM. Our study integrated the data of the NHANES population and MR genetic evidence to double‐verify the causal relationship between asthma and T2DM. The NHANES provides large‐scale and multiethnic population data that reflect the heterogeneity of the real world. Meanwhile, MR eliminates confounding factors and enhances the robustness of the conclusions, thereby avoiding the limitations of existing studies. Consequently, patients with asthma are at an elevated risk of developing T2DM, reinforcing the status of asthma as a significant risk factor.

Multiple regression analysis revealed an association between asthma and various risk factors of T2DM, including participant demographics, living conditions, lifestyle habits, intensity of physical activity, and obesity. Our study found that the incidence of T2DM was notably higher among smokers with asthma than among nonsmokers. Stapleton et al. [22] reported that both active and passive smoking contributed significantly to increased frequency and severity of asthmatic episodes. A cross‐sectional study in Kuwait highlighted environmental tobacco smoke exposure and smoking status as strong influencing factors of adolescent‐onset asthma [23]. Smoking increases the incidence of T2DM in a dose‐dependent manner [24]. Wei et al. [25] found that smokers with a genetic predisposition to T2DM or insulin resistance may have an increased risk of developing the disease. The nicotine present in tobacco can directly induce injury to pancreatic β cells, impair insulin signaling pathways, disrupt insulin receptor sensitivity, and exacerbate insulin resistance [26, 27]. In this study, obesity was also recognized as a significant factor influencing the relationship between asthma and T2DM, and asthma patients with abdominal obesity had a heightened risk of developing T2DM. Weight loss in obese T2DM patients may modestly reduce the frequency and severity of asthmatic attacks [28]. Furthermore, asthma may interact synergistically with obesity to elevate circulating inflammatory cytokine levels, thereby increasing insulin resistance [29, 30]. Wu et al. [31] reported that metabolic dysfunction could worsen asthma symptoms in obese individuals while simultaneously increasing the incidence of T2DM. We also found that patients with asthma who engaged in moderate or intense exercise exhibited a lower incidence of T2DM than those with insufficient exercise. Exercise interventions can enhance aerobic fitness, alleviate asthma symptoms, and improve the overall quality of life [32]. McLoughlin et al. [33] discovered that moderate‐to‐vigorous intensity aerobic and resistance training may be advantageous in managing moderate‐to‐severe asthma. Maurer et al. [34] suggested that children with asthma could benefit from regular exercise because elevated cytokine levels indicate an immune system primed for robust responses against various infections. Regular engagement in moderate‐to‐high intensity physical activity can also help prevent the onset of T2DM. A previous meta‐analysis provided compelling evidence supporting an inverse relationship between physical activity and the risk of developing T2DM [35]. Luo et al. [36] indicated that participation in physical activities should be encouraged for individuals at a high genetic risk for T2DM. Therefore, we propose that maintaining BMI within a healthy range, refraining from smoking, and engaging in moderate‐ or high‐intensity exercises may reduce the risk of developing T2DM among individuals with asthma.

The strength of this study lies in its national design based on data from the NHANES, which provides comprehensive documentation regarding asthma and T2DM. This approach not only offers an unbiased estimate of the causal relationship between asthma and T2DM but also enhances our understanding of this association. However, certain limitations warrant attention; these results pertain specifically to U.S. adults, potentially limiting their generalizability to other populations. Therefore, longitudinal studies should be conducted to validate the causal relationship between asthma and T2DM in future research.

## 5. Conclusions

Our findings, derived from a large cross‐sectional study utilizing NHANES database analysis, revealed a positive association between asthma and the risk of T2DM, underscoring the causal effect of asthma on the likelihood of developing this condition.

## Author Contributions

Xuemei Huang and Decheng Lu designed this research. Jinyue Meng, Jianli Huang, and Jinqun Ye performed the research. Li Liu and Cen Huang conducted statistical analyses. Xuemei Huang wrote the first draft of the manuscript. Decheng Lu revised the manuscript for intellectual content.

## Funding

This work was supported by the Research and Cultivation Project of First People’s Hospital of Nanning (Grant no. YNPY2023003), the Youth Science Foundation of Guangxi Medical University (Grant no. GXMUYSF202232), and the Nanning Qingxiu District Science and Technology Plan Project (Grant no. 2022012).

## Disclosure

All authors have reviewed and approved the final version of the manuscript.

## Ethics Statement

All NHANES protocols received approval from the Ethical Review Committee of the National Center for Health Statistics (Approval number: #2005‐06, #2011‐17).

## Conflicts of Interest

The authors declare no conflicts of interest.

## Supporting Information

Supporting Table 1: Association analysis of asthma and T2DM risk.

## Supporting information


**Supporting Information** Additional supporting information can be found online in the Supporting Information section.

## Data Availability

The datasets generated during and/or analyzed during the current study are available in the NHANES repository [https://wwwn.cdc.gov/nchs/nhanes/search/] and IEU Open‐GWAS repository [https://gwas.mrcieu.ac.uk/].
