# Supplementary material for: Association Between Asthma and the Risk of Type 2 Diabetes Mellitus: Results From NHANES 2009–2016 Data
Source: Int J Endocrinol. 2026 Jan 31;2026:4046954. doi: 10.1155/ije/4046954 (PMC12860417; doi:10.1155/ije/4046954)
Supplement: Supplementary file 1 — Supporting Information Additional supporting information can be found online in the Supporting Information section. [file IJE-2026-4046954-s001.zip › Table S1.docx]

TABLE S1: Association analysis of asthma and T2DM risk.

| Exposure | Model 1  OR（95% CI） | Model 2  OR（95% CI） | Model 3  OR（95% CI） |
| --- | --- | --- | --- |
| MCQ 035 | 2.24（1.55-3.24） | 2.26（1.55-3.31） | 1.92（1.26-2.92） |
| *P* value | 4.58E-05 | 6.70E-05 | 3.55E-03 |

Model 1 didn’t adjust for covariates; Model 2 adjusted for age, race, and gender based on Model 1; Model 3 further adjusted for marital status, educational level, family income, smoking status, drinking status, hypertension, BMI, physical activity, waist circumference, total cholesterol, high-density lipoprotein cholesterol, alanine aminotransferase, γ -glutamyl transpeptidase, serum creatinine, serum uric acid, and hemoglobin based on Model 2. T2DM: type 2 diabetes mellitus; OR: odds radio; CI: confidence interval.
